# Supplementary material for: Operational analysis for COVID-19 testing: Determining the risk from asymptomatic infections
Source: PLoS One. 2023 Feb 13;18(2):e0281710. doi: 10.1371/journal.pone.0281710 (PMC9925232; doi:10.1371/journal.pone.0281710)
Supplement: S1 File — (PDF) [file pone.0281710.s001.pdf]

Supporting information

S1 Brief review of the binomial distribution and likelihood

We use the language of coin-flipping to illustrate the ideas and let $\mathcal{B}(k, N, p) = \binom{N}{k} p^k (1-p)^{N-k}$ denote the probability of k heads in N flips. Since the outcome of each flip must be either heads or tails, the sum of these probabilities over all values of k must be 1. That is $\sum_{k=0}^{N} \mathcal{B}(k, N, p) = 1$.

The expected or mean number of heads is $\mathcal{E}(K|N, p) = Np$, and the variance in the number of heads is $Var(K|N, p) = Np(1-p)$ [49]. The coefficient of variation (standard deviation divided by the mean) in the number of flips is thus

$$\begin{aligned} CV(K|N,p) &= \tfrac{\sqrt{Var(K|N,p)}}{\mathcal{E}(K|N,p)} \\ &= \tfrac{\sqrt{Np(1-p)}}{Np} \\ &= \tfrac{1}{\sqrt{N}} \cdot \sqrt{\tfrac{1-p}{p}}, \end{aligned} \tag{S1}$$

The coefficient of variation of K scales as $1/\sqrt{N}$, which provides intuition about how test range scales with the number of tests, as in Eqn 2 of the main text.

The binomial likelihood

For the binomial likelihood, we begin with the observation of K heads in N flips and ask what inferences can be made about the probability that heads will come up on a single flip.

The likelihood of p given K and N, which we write as $\mathcal{L}(p|K, N)$ where the vertical line is used to separate the quantity about which we wish to make inference (p) and the observations (K and N), is defined to be

$$\mathcal{L}(p|K,N) = \binom{N}{k} p^k (1-p)^{N-k}. \tag{S2}$$

That is, the likelihood has the same mathematical form as $\mathcal{B}(k, N, p)$ but a different interpretation. The most salient points are: 1) p is a continuous variable ranging from 0 to 1, so that instead of summing one should integrate. For the results presented in this

paper, I discretized the interval between 0 and 1 into increments of 0.001) and replaced integration by summation. 2) Since the binomial coefficient is independent of p, we can write $\mathcal{L}(p|K,N) \propto p^k(1-p)^{N-k}$. 3) Unlike the binomial distribution that sums to 1 as k ranges between 0 and N, the binomial likelihood does not integrate/sum to 1 over p ranges from 0 to 1.

We can convert the binomial likelihood to a probability distribution by normalizing the likelihood by its weighted sum. That is, if all values of p are considered equally likely before data are collected (so that p has a uniform prior), according to Bayes' theorem of conditional probability, the probability density of p given the data, denoted by $\phi(p|K,N)$, is

$$\phi(p|K,N) = \frac{\mathcal{L}(p|K,N)}{\sum_{p'=0}^{1} \mathcal{L}(p'|K,N)}, \tag{S3}$$

where we have replaced what is more properly an integral (since p is continuous) by a sum (the form used in computations), and we use p' to reduce confusion about where p appears on the right side of Eq S3. That is, the denominator in this equation is a normalization constant.

If one had prior information about possible values of p, denoted by $\phi_0(p)$, the analogue of Eqn S3 is

$$\phi(p|K,N) = \frac{\mathcal{L}(p|K,N)\phi_0(p)}{\sum_{p'=0}^{1} \mathcal{L}(p'|K,N)\phi_0(p')}. \tag{S4}$$

Derivation of Eqn 7

For the binomial likelihood in Eqn S2, the value of p that maximizes the likelihood (the Maximum Likelihood Estimate, MLE) is $\hat{p} = K/N$ [48,49]. We will now derive Eqn 7 in the main text. We start with the likelihood of P positive tests in T total tests when the incidence rate is f (Eqn 6, repeated here for simplicity)

$$\mathcal{L}(p_+(f)|P,T) = \binom{T}{P} p_+(f)^P (1-p_+(f))^{T-P}$$

The MLE estimate for the incidence rate, $\hat{f}$ is the value of f making $\mathcal{L}(p_+(f)|P,T)$ a maximum. Since the value of a variable that maximizes a function also maximizes its logarithm, we begin by taking the logarithm of the likelihood

$$log(\mathcal{L}(p_+(f)|P,T)) = log\left[\binom{T}{P}\right] + P \cdot log(p_+(f)) + (T-P) \cdot log(1-p_+(f))$$

where we have used the mathematical fact that $log(a^z) = zlog(a)$. To find the value of f that maximizes the logarithm of the likelihood, we take the derivative of the right side and set it equal to 0. Since the first term on the right side is independent of f, its derivate is zero. For the second two terms, we use the chain rule
$\frac{d}{dx}log(y(x)) = \frac{1}{y(x)}y'(x)$ to conclude that the MLE satisfies

$$\frac{P}{p_+(\hat{f})}p'_+(\hat{f}) + \left[\frac{T-P}{1-p_+(\hat{f})}\right]p'_+(\hat{f}) = 0$$

We then factor out $p'_+(\hat{f})$ and combine the two terms with a common denominator to obtain

$$p'_+(\hat{f})\left[\frac{P}{p_+(\hat{f})} - \frac{T-P}{1-p_+(\hat{f})}\right] = p'_+(\hat{f})\left[\frac{P-Tp_+(\hat{f})}{p_+(\hat{f})(1-p_+(\hat{f}))}\right] = 0$$

which is Eqn 7 once we move the denominator outside of the brackets.

S2 Mathematical appendix.

The mean of $\hat{g}$

To compute $\mathcal{E}(\hat{g})$, we begin with

$$c_1(P_S, T_S) = \frac{(P_S/T_S) - p_{SFP}}{1 - p_{SFN} - (P_S/T_S)} \tag{S5}$$

so that

$$\begin{aligned} 1 + c_1(P_S, T_S) &= \tfrac{1-p_{SFN}-(P_S/T_S)+(P_S/T_S)-p_{SFP}}{1-p_{SFN}-(P_S/T_S)} \\ &= \tfrac{1-p_{SFN}-p_{SFP}}{1-p_{SFN}-(P_S/T_S)}. \end{aligned} \tag{S6}$$

From the far right side of Eq 23, evaluating $\mathcal{E}(\hat{g})$ requires that we compute $\mathcal{E}(T_S)$ and $\mathcal{E}(P_S)$. It is clear that $\mathcal{E}(T_S) = T(f_t + g_t)$ since the probability of testing a symptomatic individual is $f_t + g_t$.

To evaluate $\mathcal{E}(P_S)$, we use the rule of conditional expectation,

$$\begin{aligned} \mathcal{E}(P_S) &= \mathcal{E}_{T_S}\big[\mathcal{E}(P_S|T_S)\big] \\ &= \mathcal{E}_{T_S}\big[T_S \cdot \tfrac{f_t(1-p_{SFN})+g_t p_{SFP}}{f_t+g_t}\big] \\ &= T\big[f_t(1-p_{SFN}) + g_t p_{SFP}\big] \end{aligned} \tag{S7}$$

Using these expectations in Eq 24, we conclude

$$\mathcal{E}(\hat{g}) = \frac{1}{T} \cdot \frac{1}{1 - p_{SFN} - p_{SFP}} \cdot \Big[T(f_t + g_t)(1 - p_{SFN}) - T\big[f_t(1 - p_{SFN}) + g_t p_{SFP}\big]\Big], \text{ so that}$$
$$\mathcal{E}(\hat{g}) = \frac{1}{T} \cdot \frac{1}{1 - p_{SFN} - p_{SFP}} \cdot \Big[T g_t(1 - p_{SFN} - p_{SFP})\Big] \text{ from which}$$
$$\mathcal{E}(\hat{g}) = g_t. \tag{S8}$$

For completeness, I now give the expectations P_S^2, T_S^2, and $T_S P_S$:

$$\mathcal{E}(P_S^2) = p_S p_{S+} T - p_{S+}^2 p_S T + p_{S+}^2 \Big[T p_S + T p_S^2 (T-1) \Big],$$
$$\mathcal{E}(T_S^2) = T p_S + T p_S^2 (T-1), \text{ and}$$
$$\mathcal{E}(T_S P_S) = p_{S+} \mathcal{E}(T_S^2). \tag{S9}$$

These computations are based on the rule of total expectation that if X and Y are random variables,

$$\mathcal{E}(X) = \mathcal{E}_Y[\mathcal{E}(X|Y)], \tag{S10}$$

and that if K has the binomial distribution $\mathcal{B}(\cdot|, N, p)$, the mean and variance of K are Np and $Np(1-p)$, respectively. Since by definition $VarK = \mathcal{E}(K^2) - \mathcal{E}(K)^2$, we conclude

$$\begin{aligned} \mathcal{E}(K^2) = & \quad Var(K) + \mathcal{E}(K)^2 = Np(1-p) + (Np)^2 \\ = & \quad Np - Np^2 + p^2 N^2 \\ = & \quad Np + Np^2(N-1), \end{aligned} \tag{S11}$$

Now, $T_S \sim \mathcal{B}(\cdot|T, p_S)$, where p_S is given by Eq 12, and given T_S, $P_S \sim \mathcal{B}(\cdot|T_S, p_{S+})$, where p_{S+} is given by Eq 13. Thus, we immediately conclude

$$\mathcal{E}(T_S^2) = T p_S + T p_S^2 (T-1). \tag{S12}$$

Using the rule of total expectation,

$$\begin{aligned}\mathcal{E}(T_S P_S) = {} & \mathcal{E}_{T_S}\Big[\mathcal{E}(T_S P_S|T_S)\Big] \\ = {} & \mathcal{E}_{T_S}\Big[T_S\mathcal{E}(P_S|T_S)\Big] \\ = {} & \mathcal{E}_{T_S}\Big[T_S p_{S+} T_S\Big] \\ = {} & p_{S+}\mathcal{E}(T_S^2). \end{aligned} \tag{S13}$$

We similarly evaluate

$$\begin{aligned}\mathcal{E}(P_S^2) = {} & \mathcal{E}_{T_S}\Big[\mathcal{E}(P_S^2|T_S)\Big] \\ = {} & \mathcal{E}_{T_S}\Big[T_S p_{S+} + T_S^2 p_{S+}^2 - T_S p_{S+}^2\Big] \\ = {} & p_S p_{S+} T - p_{S+}^2 p_S T + p_{S+}^2\Big[T p_S + T p_S^2 (T-1)\Big]. \end{aligned} \tag{S14}$$

The mean of $\hat{\rho}$

We begin with Eq 21, rewritten as

$$\hat{\rho} = \frac{(1-\hat{f}-\hat{g})}{(1-p_{AFN}-p_{AFP})\hat{f}}\left[\frac{P-P_S-p_{AFP}(T-T_S)}{T-T_S}\right]. \tag{S15}$$

We first show that $\hat{f}+\hat{g} = T_S/T$ (as intuition suggests it should be). From Eqs 18 and 19,

$$\begin{aligned}\hat{f}+\hat{g} = {} & \hat{g}\left[1+\tfrac{(P_S/T_S)-p_{SFP}}{1-p_{SFN}-(P_S/T_S)}\right] \\ = {} & \hat{g}\left[1+\tfrac{P_S-p_{SFP}T_S}{(1-p_{SFN})T_S-P_S}\right] \\ = {} & \hat{g}\left[\tfrac{(1-p_{SFN})T_S-P_S+P_S-p_{SFP}T_S}{(1-p_{SFN})T_S-P_S}\right] \\ = {} & \hat{g}\left[\tfrac{T_S(1-p_{SFN}-p_{SFP})}{(1-p_{SFN})T_S-P_S}\right]. \end{aligned} \tag{S16}$$

Now again use Eq 19 to obtain

$$\begin{aligned}\hat{f}+\hat{g} = &\quad \tfrac{1}{T}\cdot\left[\tfrac{T_S(1-p_{SFN})-P_S}{1-p_{SFN}-p_{SFP}}\right]\cdot\left[\tfrac{T_S(1-p_{SFN}-p_{SFP})}{(1-p_{SFN})T_S-P_S}\right]\\ = &\quad \tfrac{T_S}{T}.\end{aligned} \tag{S17}$$

Consequently, Eq S15 becomes

$$\begin{aligned}\hat{\rho} = &\quad \tfrac{(1-(T_S/T)}{(1-p_{AFN}-p_{AFP})\hat{f}}\left[\tfrac{P-P_S-p_{AFP}(T-T_S)}{T-T_S}\right]\\ = &\quad \tfrac{(T-T_S)}{T(1-p_{AFN}-p_{AFP})\hat{f}}\left[\tfrac{P-P_S-p_{AFP}(T-T_S)}{T-T_S}\right]\\ = &\quad \tfrac{1}{T(1-p_{AFN}-p_{AFP})\hat{f}}\Big[P-P_S-p_{AFP}(T-T_S)\Big].\end{aligned} \tag{S18}$$

To simplify the notation in the rest of this section, we let $x=\hat{f}, y=P-P_S$ and $z=T-T_S$ and as before $c_1=1-p_{AFN}-p_{AFP}$ so that Eq S18 becomes

$$\hat{\rho}(x,y,z)=\frac{1}{Tc_2}\cdot\frac{1}{x}(y-p_{AFP}z). \tag{S19}$$

The delta-method involves Taylor expanding Eq S19 to second order in the variables around their means and then taking expectations (see Hilborn and Mangel [50, pp. 58–59] for a general explanation; see Mangel and Brown, [30, pp. 26–27] for application to COVID-19 testing). Using subscripts to denote partial derivatives, overbars to represent the means of x, y, and z, $Cov(i,j)$ $[i,j=x,y,x]$ the covariance between the random variables i and j, with $Cov(i,i)$ understood to be the variance of random variable i, we have

$$\mathcal{E}(\hat{\rho}(x,y,z))=\hat{\rho}(\overline{x},\overline{y},\overline{z})+\frac{1}{2}\sum_{i,j=x,y,z}\hat{\rho}_{ij}(\overline{x},\overline{y},\overline{z})Cov(i,j). \tag{S20}$$

We first show that

$$\hat{\rho}(\overline{x}, \overline{y}, \overline{z}) = \rho_t \tag{S21}$$

(i.e., to first order the point estimate for the ratio of asymptomatic to symptomatic infections is the true value of the ratio) by recalling that we have already shown that $\mathcal{E}(\hat{f}) = f_t$, so that $\overline{x} = f_t$. Similarly, since $\mathcal{E}(T - T_S) = T(1 - f_t - g_t)$, $\overline{z} = T(1 - f_t - g_t)$.

We compute $\overline{y}$ by using the rule of conditional expectation for $P - P_S$,

$$\begin{aligned}
\mathcal{E}(P - P_S) = & \quad \mathcal{E}_{T_S, P_S}[\mathcal{E}(P - P_S | T_S, P_S)] \\
= & \quad \mathcal{E}_{T_S}[(T - T_S) p_{A+}] \\
= & \quad \mathcal{E}_{T_S}[(T - T_S) \tfrac{f_t \rho_t (1 - p_{AFN}) + (1 - g_t - f_t(1 + \rho_t)) p_{AFP}}{1 - f_t - g_t}] \\
= & \quad T(1 - f_t - g_t)[\tfrac{f_t \rho_t (1 - p_{AFN}) + (1 - g_t - f_t(1 + \rho_t)) p_{AFP}}{1 - f_t - g_t}] \\
= & \quad T[\rho_t f_t (1 - p_{AFN} - p_{AFP}) + (1 - f_t - g_t) p_{AFP}],
\end{aligned} \tag{S22}$$

so that

$$\hat{\rho}(\overline{x}, \overline{y}, \overline{z}) = \tfrac{T}{T c_2 f_t}[\rho_t f_t (1 - p_{AFN} - p_{AFP}) + (1 - f_t - g_t) p_{AFP} - p_{AFP}(1 - f_t - g_t)] = \rho_t$$

Since

$$\hat{\rho}(x, y, z) = \frac{1}{T c_2} \cdot \frac{1}{x} (y - p_{AFP} z), \tag{S23}$$

direct inspection shows that the three first-order partial derivatives are

$$
\begin{aligned}
\hat{\rho}_x &= -\frac{1}{x}\hat{\rho} \\
\hat{\rho}_y &= \frac{1}{Tc_2}\cdot\frac{1}{x} \\
\hat{\rho}_z &= -\frac{p_{AFP}}{Tc_2}\cdot\frac{1}{x}
\end{aligned}
\tag{S24}
$$

Because $\hat{\rho}_y$ and $\hat{\rho}_z$ are independent of y and z respectively, we conclude that $\hat{\rho}_{yy} = \hat{\rho}_{zz}$=0. The remaining second partial derivatives are determined by inspection:

$$
\begin{aligned}
\hat{\rho}_{xx} &= \frac{1}{x^2}\hat{\rho} - \frac{1}{x}\hat{\rho}_x = \frac{2\hat{\rho}}{x^2} \\
\hat{\rho}_{xy} = \hat{\rho}_{yx} &= -\frac{1}{Tc_2}\cdot\frac{1}{x^2} \\
\hat{\rho}_{xz} = \hat{\rho}_{yz} &= \frac{p_{AFP}}{Tc_2}\cdot\frac{1}{x^2}.
\end{aligned}
\tag{S25}
$$

Using these equations and returning to the original variables $x = \hat{f}, y = P - P_s$, and $z = T - T_s$, we conclude

$$
\mathcal{E}(\hat{\rho}) = \rho_t + \frac{1}{2}\left[\frac{2\rho_t}{f_t^2}Var(\hat{f}) - \frac{2}{Tc_2 f_t^2}Cov(\hat{f}, P - P_S) + \frac{2p_{AFP}}{Tc_2 f_t^2}Cov(\hat{f}, T - T_S)\right], \tag{S26}
$$

which is Eq 29 of the main text.

S3 Sensitivity analysis when there is information on symptoms

In this Supplementary information, I show results analogous to Figures 7, 9 and 10, and 11 for different values of the true state of the world and two values of test numbers. In particular:

$f_t = 0.05, g_t = 0.04, \rho_t = 1.5, T = 1500$ or 3000 [these are the base case parameters];

$f_t = 0.03, g_t = 0.04, \rho_t = 1.5, T = 1500$ or 3000;

$f_t = 0.01, g_t = 0.04, \rho_t = 1.5, T = 1500$ or 3000;

$f_t = 0.05, g_t = 0.02, \rho_t = 1.5, T = 1500$ or 3000;

$f_t = 0.05, g_t = 0.04, \rho_t = 2.0, T = 1500$ or 3000;

$f_t = 0.05, g_t = 0.04, \rho_t = 1.0, T = 1500$ or 3000;

$f_t = 0.05, g_t = 0.04, \rho_t = 2.5, T = 1500$ or 3000; and

$f_t = 0.07, g_t = 0.04, \rho_t = 1.5, T = 1500$ or 3000.

In this sensitivity analysis, the test data are not the mean test data but a random sample based on the true values (shown as a white dot in the lower left panels, as in Figure 10 in the main text).

$f_t = 0.05, g_t = 0.04, \rho_t = 1.5, T = 1500$ or 3000 (Base Case)

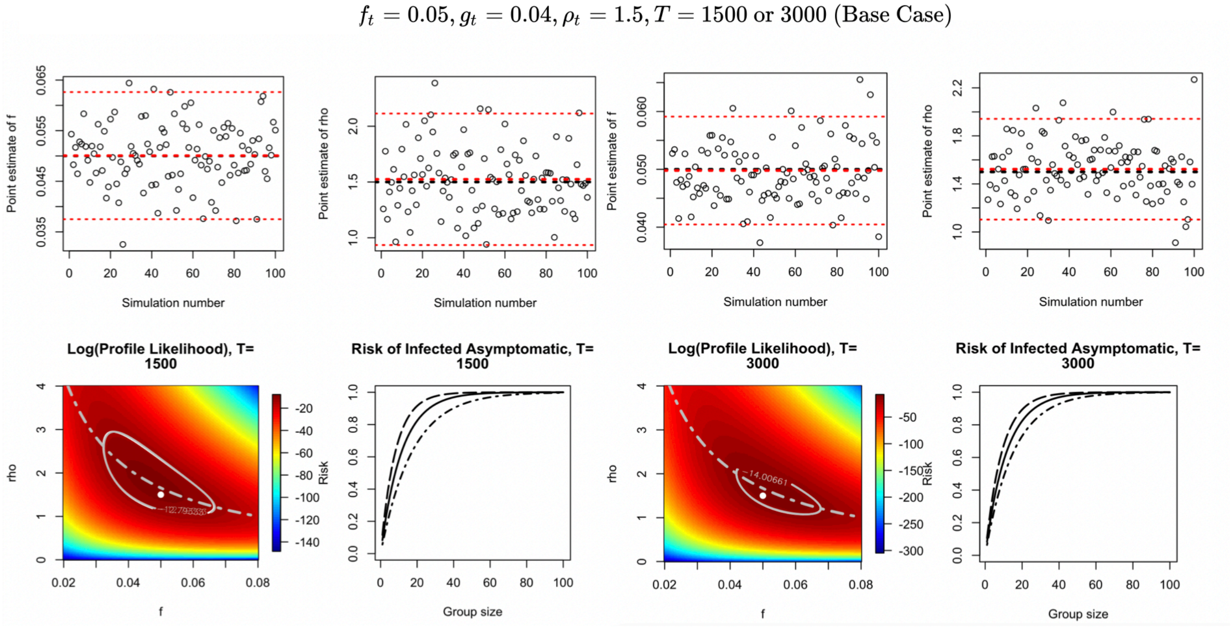

$f_t = 0.03, g_t = 0.04, \rho_t = 1.5, T = 1500$ or 3000

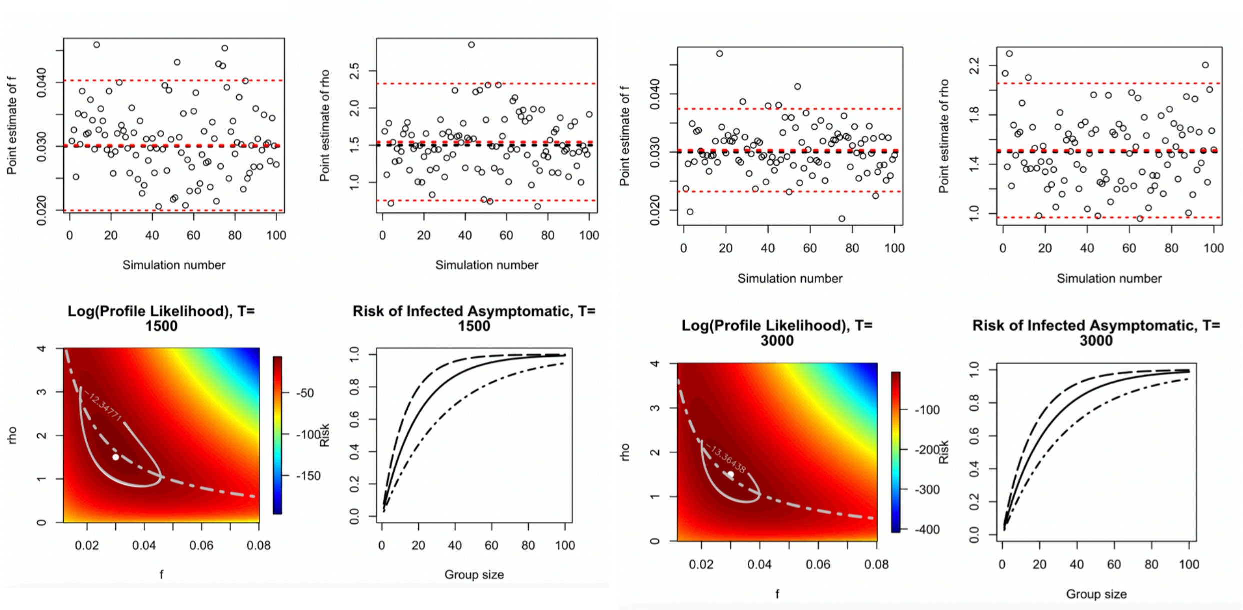

$f_t = 0.01, g_t = 0.04, \rho_t = 1.5, T = 1500$ or 3000

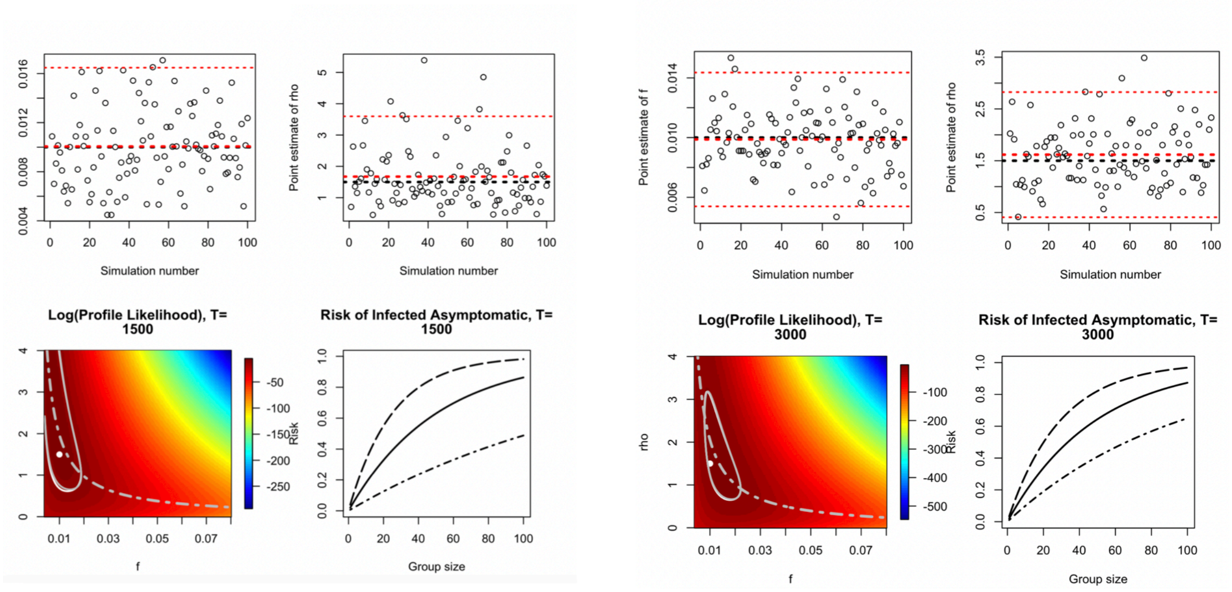

$f_t = 0.05, g_t = 0.02, \rho_t = 1.5, T = 1500$ or 3000

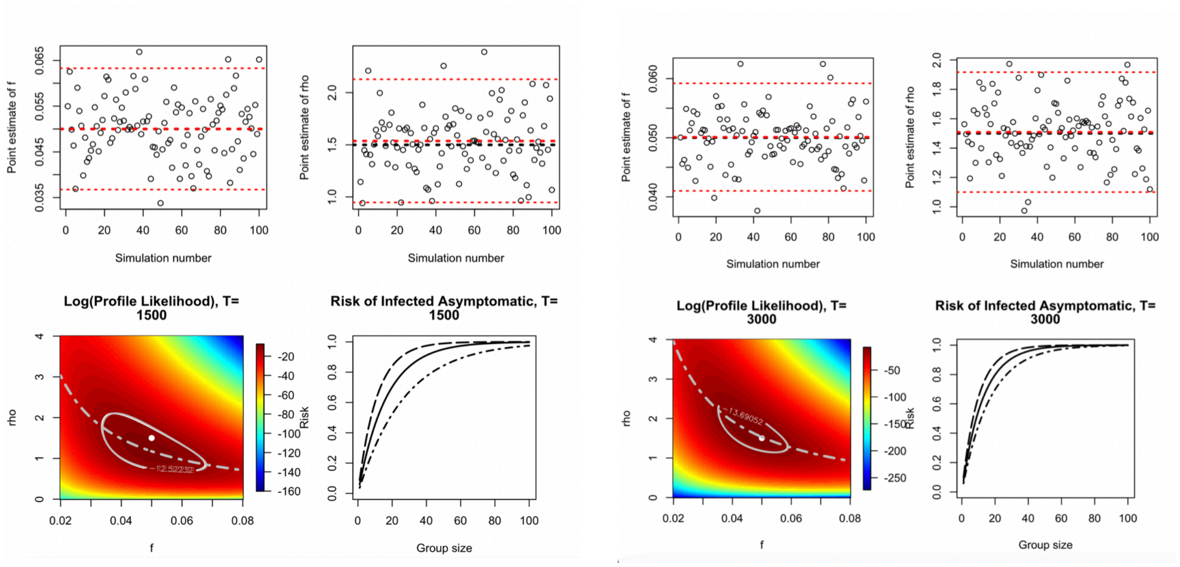

$$f_t = 0.05, g_t = 0.04, \rho_t = 2.0, T = 1500 \text{ or } 3000$$

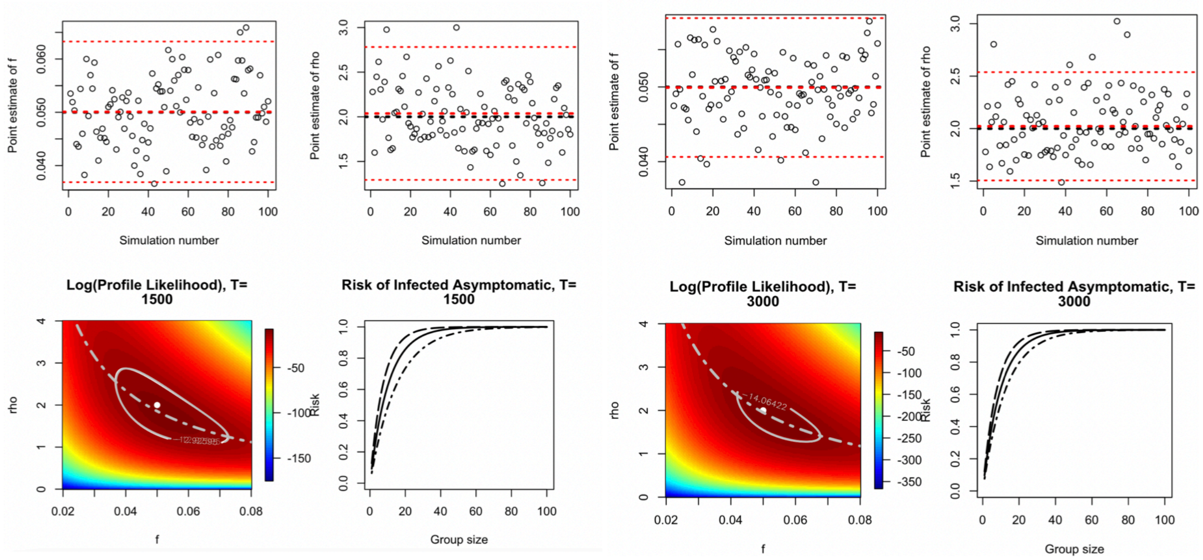

$$f_t = 0.05, g_t = 0.04, \rho_t = 1.0, T = 1500 \text{ or } 3000$$

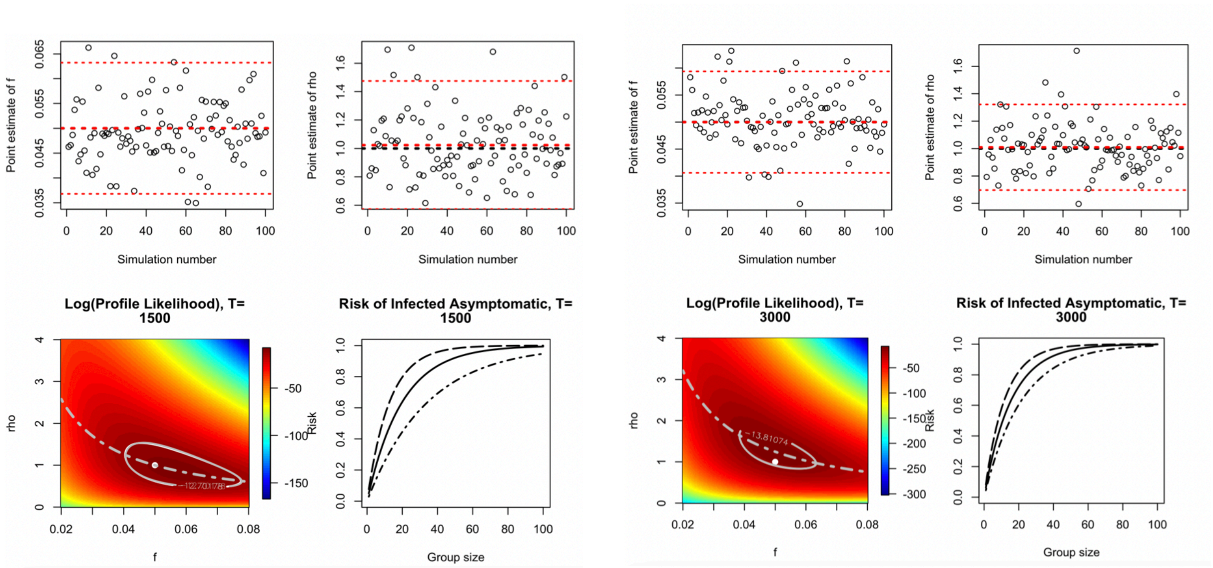

$$f_t = 0.05, g_t = 0.04, \rho_t = 2.5, T = 1500 \text{ or } 3000$$

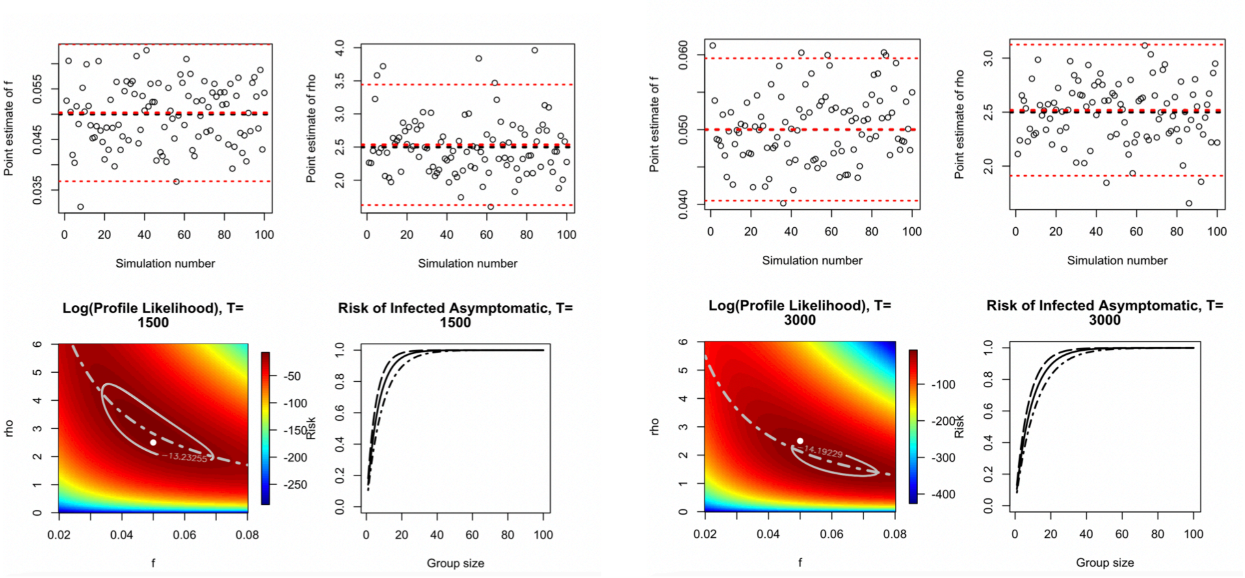

$$f_t = 0.07, g_t = 0.04, \rho_t = 1.5, T = 1500 \text{ or } 3000$$

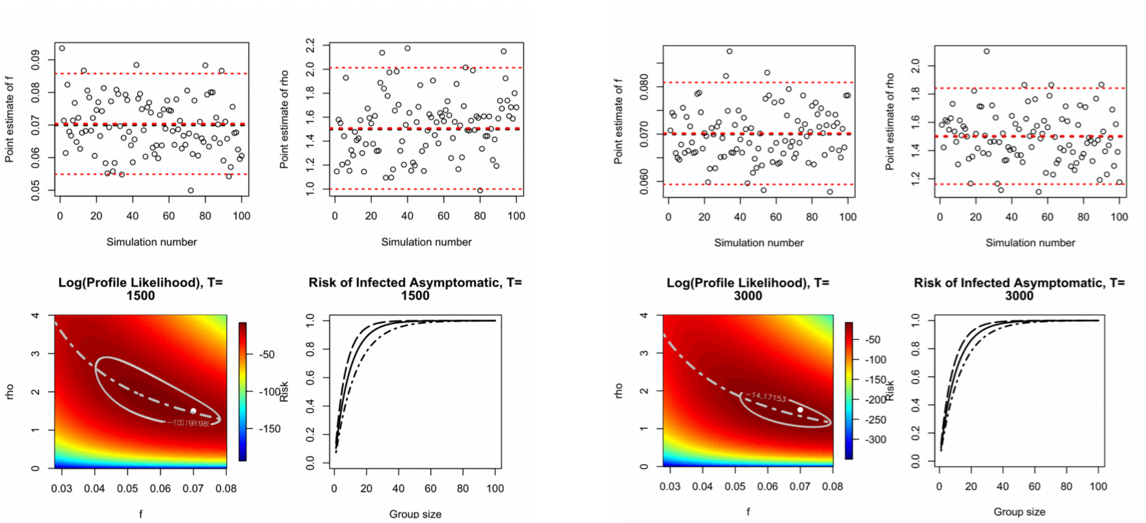

S4 Codes that generate the results in the main text and sensitivity analysis

These codes are separated as: 1) code to generate the results shown in Figures 1 and 2, in which there is no information on symptoms and positivity exceeds the probability of a false positive test; 2) code to generate the results shown in Figures 4-6, in which there is no information on symptoms and positivity is greater than 0 but less than the probability of a false positive test; and 3) code to generate the results shown in Figures 7-11 and the Supplementary figures, in which there is information on symptoms.

When preparing this supplementary information, I focused on the accessibility and clarity of the codes rather than their sleekness or efficiency (although none of them take very long to run). That is, these codes are aimed for the neophyte rather than seasoned programmer, since the latter individuals can always soup them up.

1) Code to generate Figures 1 and 2 (no information on symptoms and positivity exceeds the probability of a false positive test)

```
#This code generates Figures 1 and 2 for the case in which there is no information
#on symptoms
#Figure 1 is a simple deterministic calculation based on Eqn 3 of the main text (so that
# the same results are always generated)
#Figure 2 is simulation (so that results will vary as the simulation is repeated)


library(fields)
require(graphics)

#***************************** Figure 1 ****************************************
#**********************************************************************

#This is code to produce Figure 1.  It is a deterministic computation based on Eqn 3 of
#the main text

Group_size=seq(from=2,to=100,length=99)
f_t=0.05
Risk=1-(1-f_t)^Group_size

plot(Group_size,Risk,type="l",lwd=3,xlab="Group size",ylab="Risk",ylim=c(0,1))


#***************************** Figure 2 ****************************************
#**********************************************************************


#************************* Part 1: Basic structure ***************
```

```
N_sim=100
T_S=rep(0,N_sim) #symptomatics in the sample
P_S=rep(0,N_sim) #number of symptomatics testing positive
P=rep(0,N_sim)
f_hat=rep(0,N_sim)
f_hat_min=rep(0,N_sim)
f_hat_max=rep(0,N_sim)
Range=rep(0,N_sim)
p_pos_n=rep(0,N_sim)
rho_hat=rep(0,N_sim)
g_hat=rep(0,N_sim)
rho_hat_1=rep(0,N_sim)

T=2500 #number of tests



T_S=rep(0,N_sim) #symptomatics in the sample
P_S=rep(0,N_sim) #number of symptomatics testing positive
P=rep(0,N_sim)
f_hat=rep(0,N_sim)
rho_hat=rep(0,N_sim)
g_hat=rep(0,N_sim)
rho_hat_1=rep(0,N_sim)

#************** Part 2: Surface positivity is not incidence3 rate, ala  References 29 and 
30,************
# This section is not included in the paper (consider it a bonus section), but allows one to see the
#difference between surface positivity and  and the estimate in Eqn 3 of the main text

f_t=0.05  # antigen posiive

p_FN =0.25 #probability of a false negative  -- Figure 13 of Mangel and Brown
p_FP=0.05 #probability of a false positive -- Figure 13 of Mangel and Brown

p_d_s_test=toString(T)
p_d_s_1=toString(p_FN)
p_d_s_2=toString(p_FP)
leg=cbind("tests, p_FN, p_FP = ",p_d_s_test,p_d_s_1,p_d_s_2)

Pos_test=rep(0,N_sim)
Surface_pos=rep(0,N_sim)

#Now lets look at the simplified version
```

```
p_pos=f_t*(1-p_FN) + (1-f_t)*p_FP

Pos_test=rbinom(N_sim,T,p_pos)
Surface_pos=Pos_test/T
f_hat=(Surface_pos-p_FP)/(1-p_FN-p_FP)

quartz()
par(mfrow=c(1,2))

plot(pch=19,Surface_pos,xlab="Simulation number",ylab="Surface
positivity",main=leg,ylim=c(0.8*f_t,0.1))
abline(h=f_t,lty=3)
surface_error_mean=mean(Surface_pos-f_t)
surface_sd=sd(Surface_pos-f_t)

plot(pch=19,f_hat,xlab="Simulation number",ylab="f_hat",main=leg,ylim=c(0,0.08))
abline(h=f_t,lty=3)
f_hat_error_mean= mean(f_hat-f_t)
f_hat_sd= sd(f_hat-f_t)

#range calculations -- not plotted here (or used in the main text)

p_pos_n=f_hat*(1-p_FN) + (1-f_hat)*p_FP
for(n in 1:N_sim)
{
  numerator=p_pos_n[n]*(1-p_pos_n[n])
  denominator=T*(1-p_FN-p_FP)^2

  Range[n]=3.92*sqrt(numerator/denominator)
  f_hat_min[n]=f_hat[n]-0.5*Range[n]
  f_hat_max[n]=f_hat[n]+0.5*Range[n]
}


#************** Part 3: Generate Figure 2 of the main text,************
#************* Remember that thes are simulations, so that results will vary quantitatively
#*from replicate to replicate but not qualitatively
I_h=199
h=seq(from=2, to=200,length=I_h)
R_t=rep(0,I_h)
R_t=1-(1-f_t)^h



R_hat=array(0,c(N_sim,I_h))
R_hat_min=array(0,c(N_sim,I_h))
```

```
R_hat_max=array(0,c(N_sim,I_h))


R_acc=seq(from=0.1, to =0.9,length=I_h)
h_acc_t=rep(0,I_h)
h_hat=array(0,c(N_sim,I_h))
h_hat_min=array(0,c(N_sim,I_h))
h_hat_max=array(0,c(N_sim,I_h))





#make  simulated risk curves
for(n in 1:N_sim)
{
  R_hat[n,]=1-(1-f_hat[n])^h
  R_hat_min[n,]=1-(1-f_hat_min[n])^h
  R_hat_max[n,]=1-(1-f_hat_max[n])^h
}


#now the group size calculation

# plot(h,R_t,type="l",lwd=4,xlab="Group size",ylab="Risk",xlim=c(0,100))
# for(n in 1:N_sim)
# {
#   lines(h,R_hat[n,],col=n+1)
# }


h_acc_t=log(1-R_acc)/log(1-f_t)

for(n in 1:N_sim)
{
  h_hat[n,]=log(1-R_acc)/log(1-f_hat[n])
  h_hat_min[n,]=log(1-R_acc)/log(1-f_hat_min[n])
  h_hat_max[n,]=log(1-R_acc)/log(1-f_hat_max[n])
}

y_max=1.1*max(h_hat)


quartz()
par(mfrow=c(4,4))
for(n in 1:16)
```

```
{
 p_d_s_test=toString(T)
 p_d_s_positivity=toString(Surface_pos[n])

 p_d_s_positivity=toString(round(10000*Surface_pos[n])/10000)
 leg=cbind("Surface positivity=",p_d_s_positivity)
 plot(R_acc,h_acc_t,type="l",lwd=2,xlab="Acceptable risk",ylab="Group size",lty=3,main=leg)
  lines(R_acc,h_hat[n,])
  lines(R_acc,h_hat_min[n,],col=4)
  lines(R_acc,h_hat_max[n,],col=6)
}
```

}

2) Code to generate the results shown in Figures 4-6 (no information on symptoms and positivity is greater than 0 but less than the probability of a false positive test)

```
#This code generates the results shown in Figures 4, 5 and 6 -- for the case in which
#there is no information on symptoms and positivity (P/T) is less than the probability
#of a false positive test (p_FP)


#test errors
p_FN=0.25
p_FP=0.05

#true value of infected fraction
I_f=2000
f=seq(from=0.0, to = 0.1, length=I_f)
p_pos=rep(0,I_f)
p_pos=f*(1-p_FN)+(1-f)*p_FP

#********************* Code to generate Figure 4 ***************
T=100
P=c(1.5*p_FP*T,1.2*p_FP*T,1.05*p_FP*T,0.8*p_FP*T)
f_hat=rep(0,4)
for(i_p in 1:4)
{
f_hat[i_p]=max(0,((P[i_p]/T)-p_FP)/(1-p_FN-p_FP))
}


LL=array(0,c(4,I_f))
for(i_p in 1:4)
{
 LL[i_p,]=P[i_p]*log(p_pos)+(T-P[i_p])*log(1-p_pos)
}


#quartz()
#par(mfrow=c(2,2))

for(i_p in 1:4)
{
 pds=toString(P[i_p]/T)
 leg=cbind("Positivity = ",pds)
plot(f,LL[i_p,],type="l",lwd=3,xlab="Incidence rate",ylab="Log-likelihood",main=leg)
abline(v=f_hat[i_p],lty=3)
}
```

```
#***************** Code to generate Figure 5 ******************

pds=toString(p_FP)

pos=0.5 #positivity must be less than p_FP so set it as a fraction of p_FP. Code needs to
#be run multiple times to generate the figure
pos=0.25
pos=0.125


I_p=50
P=rep(0,I_p)
for(i_p in 1:I_p)
{
  P[i_p]=2*i_p
}
T=rep(0,I_p) #now choose the number of tests so that we get positivity = pos*p_FP
T=P/(pos*p_FP)
T=round(T) #must be an integer so round, and check

pds1=toString(pos*p_FP)

leg=cbind("p_FP and positivity are ",pds,pds1)


L=array(0,c(I_p,I_f)) #binomial likelihood
LL=array(0,c(I_p,I_f)) #binomial likelihood
phi=array(0,c(I_p,I_f)) #normalized likelihood
f_H=rep(0,I_p)
TR=rep(0,I_p)

#make the likelihood
for(i_p in 1:I_p)
{
  for(i_f in 1:I_f)
    {
    L[i_p,i_f] =dbinom(P[i_p], T[i_p], p_pos[i_f], log = FALSE)
  }
  #assume a uniform prior to convert to a probability density (i,e. normalized likelihood)
  phi[i_p,]=L[i_p,]/sum(L[i_p,])
}
```

```
plot(f,phi[I_p,],type="l",lwd=3,xlim=c(0,0.01),main=leg,xlab="Incidence
rate",ylab="Normalized likelihood",col="white")
lines(f,phi[I_p,],lwd=3,col=1)
lines(f,phi[3*I_p/4,],lwd=3,col=2)
lines(f,phi[I_p/2,],lwd=3,col=3)
lines(f,phi[I_p/4,],lwd=3,col=4)
pds1=toString(T[I_p])
pds2=toString(T[3*I_p/4])
pds3=toString(T[I_p/2])
pds4=toString(T[I_p/4])
legend(0.003,0.2, legend=c(pds1,pds2,pds3,pds4),
      col=c(1,2,3,4), lty=1, lwd=2, cex=0.7)

#************************* Code to generate Figure 6 [the range] ******************
for(i_p in 1:I_p)
{
sum=phi[i_p,1]
f_H=f[1]
i_f=1
while(sum<=0.95)
  {
i_f=i_f+1
sum=sum+phi[i_p,i_f]
}
f_H[i_p]=f[i_f]
print(f_H[i_p])
TR[i_p]=f_H[i_p]
}


plot(T,TR,type="l",xlab="Number of tests",ylab="95% CI",main=leg,lwd=3)
```

3) Code to generate the results shown in Figures 7-11 and the Supplementary figures, in which there is information on symptoms.

```
#This code generates the results shown in Figures 7-11 of the main text and
#the supplementary figures (with modification of the paramater values characterizing the true
#state of nature, test numbers, and [if desired] the number of simulations)

#Recall that these are simulation methods, so that results will vary from replicate to
#replicate.

library(fields)
require(graphics)

#*********************** Part 1: True state of nature ******************
#Base case parameters as in the text
f_t=0.05  #symptomatic, antigen posiive base case
g_t=0.04 #symptomatic, antigen negative base case
rho_t=1.5 #ratio of asymptomatic to symptomatic antigen positive base case

T=1500 #number of tests
N_sim=1000

T_S=rep(0,N_sim) #symptomatics in the sample
P_S=rep(0,N_sim) #number of symptomatics testing positive
P=rep(0,N_sim)
f_hat=rep(0,N_sim)
rho_hat=rep(0,N_sim)
g_hat=rep(0,N_sim)
rho_hat_1=rep(0,N_sim)



#********************************** Part 2: Make the relevant data and show it (Figure
7) ****
#****************** (the lower right  hand panel in Figure 7 is obtained by changing the
# y axis on the lower left hand panel)***********************

p_SFN=0.25
p_SFP=0.03
p_AFN=0.5
p_AFP=0.003

pr_S =f_t+g_t #probability test a symptomatic individual
```

```
#probability of a positive test
pr_p=f_t*(1-p_SFN)+ g_t*p_SFP +rho_t*f_t*(1-p_AFN)+(1-g_t-f_t*(1+rho_t))*p_AFP

#probability of a positive test given symptomatic
pr_Sp=(f_t*(1-p_SFN)+g_t*p_SFP)/(f_t+g_t)

#prob of a positive test given asymptomatic
pr_Ap=(rho_t*f_t*(1-p_AFN)+(1-g_t-f_t*(1+rho_t))*p_AFP)/(1-f_t-g_t)

#ready to make the data
for(n in 1:N_sim)
{
  T_S[n]=rbinom(1,T,pr_S)
  P_S[n]=rbinom(1,T_S[n],pr_Sp)
  P_asympt=rbinom(1,T-T_S[n],pr_Ap)
  P[n]=P_S[n]+P_asympt
}

#now make the point estimates
for(n in 1:N_sim)
{


  #follow the new notation in the document
  numerator=(P_S[n]/T_S[n])-p_SFP
  denominator=1-p_SFN-(P_S[n]/T_S[n])
  c_1=numerator/denominator
  g_hat[n]=T_S[n]/(T*(1+c_1))
  f_hat[n]=c_1*g_hat[n]

  #get rho_hat from Eqn 14 by multiplying through  1-f_hat-g_hat
  b_1=(1-f_hat[n]-g_hat[n])*p_AFP
  d_1=(P[n]-P_S[n])*(1-f_hat[n]-g_hat[n])/(T-T_S[n])
  c_2=1-p_AFN-p_AFP
  rho_hat[n]=(1/(c_2*f_hat[n]))*(d_1-b_1 )
}

#Look at the data
quartz()
par(mfrow=c(2,2))

plot(f_hat[1:100],xlab="Simulation number",ylab="Point estimate of f")
abline(h=f_t,lty=3,lwd=3)
abline(h=mean(f_hat),lty=3,col=2,lwd=3)
abline(h=mean(f_hat)+1.96*sd(f_hat),lty=3,col=2,lwd=2)
abline(h=mean(f_hat)-1.96*sd(f_hat),lty=3,col=2,lwd=2)
```

```
plot(rho_hat[1:100],xlab="Simulation number",ylab="Point estimate of rho")
abline(h=rho_t,lty=3,lwd=3)
abline(h=mean(rho_hat),lty=3,col=2,lwd=3)
abline(h=mean(rho_hat)+1.96*sd(rho_hat),lty=3,col=2,lwd=2)
abline(h=mean(rho_hat)-1.96*sd(rho_hat),lty=3,col=2,lwd=2)

plot(g_hat[1:100],xlab="Simulation number",ylab="Point estimate of g")
abline(h=g_t,lty=3,lwd=3)
abline(h=mean(g_hat),lty=3,col=2,lwd=3)
abline(h=mean(g_hat)+1.96*sd(g_hat),lty=3,col=2,lwd=2)
abline(h=mean(g_hat)-1.96*sd(g_hat),lty=3,col=2,lwd=2)

#errors and relative errors
f_hat_error=(mean(f_hat)-f_t)/f_t
f_hat_error=floor(10000*f_hat_error)/10000
g_hat_error=(mean(g_hat)-g_t)/g_t
g_hat_error=floor(10000*g_hat_error)/10000
rho_hat_error=(mean(rho_hat)-rho_t)/rho_t
rho_hat_error=floor(10000*rho_hat_error)/10000
datum=cbind("Relative mean errors f, rho, g",f_hat_error,rho_hat_error,g_hat_error)

#how many outside of the naive 95% Gaussian interval
N_f_out=0
N_g_out=0
N_rho_out=0
for(n in 1:N_sim)
{
  if(f_hat[n]>mean(f_hat)+1.96*sd(f_hat)){N_f_out=N_f_out+1}
  if(f_hat[n]<mean(f_hat)-1.96*sd(f_hat)){N_f_out=N_f_out+1}
}

for(n in 1:N_sim)
{
  if(g_hat[n]>mean(g_hat)+1.96*sd(g_hat)){N_g_out=N_g_out+1}
  if(g_hat[n]<mean(g_hat)-1.96*sd(g_hat)){N_g_out=N_g_out+1}
}

for(n in 1:N_sim)
{
  if(rho_hat[n]>mean(rho_hat)+1.96*sd(rho_hat)){N_rho_out=N_rho_out+1}
  if(rho_hat[n]<mean(rho_hat)-1.96*sd(rho_hat)){N_rho_out=N_rho_out+1}
}
```

```
datum_out=cbind("Fraction outside 95%CI f, rho,
g",N_f_out/N_sim,N_rho_out/N_sim,N_g_out/N_sim)
print(datum)
print(datum_out)



#************************* Generate the results shown in Figures 7-11 and the
Supplementary Figures
#*This is set up to choose between the mean data and randomly generated data

#Mean data are known without simulation, based on the true state of the world
T_S_bar=(f_t+g_t)*T
P_S_bar=pr_Sp*T_S_bar
P_A_bar=(T-T_S_bar)*pr_Ap
P_bar=P_S_bar+P_A_bar

#Number of positive tests have to be integers
P_S_bar=round(P_S_bar)
P_bar=round(P_bar)

data_choice=0 #use the mean values
#data_choice=1 #randomly generate data -- results will vary from replicate to replicate


if(data_choice>0)
{
#now draw them randomly but do not change the notation just yet
T_S_bar=rbinom(1,T,pr_S)
P_S_bar=rbinom(1,T_S_bar,pr_Sp)
P_A_bar=rbinom(1,T-T_S_bar,pr_Ap)
P_bar=P_S_bar+P_A_bar
}

p_d_T=toString(T)
p_d_T_S=toString(T_S_bar)
p_d_P_S=toString(P_S_bar)
p_d_P=toString(P_bar)

datum_bar=cbind("T,T_S, P_S,  P",p_d_T,p_d_T_S,p_d_P_S,p_d_P)
print(datum_bar)


#****************** Likelihood of the symptomatic data (Figure 8) **************
```

```
numerator=(P_S_bar/T_S_bar)-p_SFP
denominator=1-p_SFN-(P_S_bar/T_S_bar)
c_1=numerator/denominator
g_hat_bar=T_S_bar/(T*(1+c_1))
f_hat_bar=c_1*g_hat_bar

b_1=(1-f_hat_bar-g_hat_bar)*p_AFP
d_1=(P_bar-P_S_bar)*(1-f_hat_bar-g_hat_bar)/(T-T_S_bar)
c_2=1-p_AFN-p_AFP
rho_hat_bar=(1/(c_2*f_hat_bar))*(d_1-b_1 )

f_min=0.4*f_t
#make this smaller
f_min=0.1*f_t
f_max=1.6*f_t
f_min=floor(10000*f_min)/10000
f_max=floor(1000*f_max)/1000

g_min=0.4*g_t
g_max=1.6*g_t
g_min=floor(1000*g_min)/1000
g_max=floor(1000*f_max)/1000

f_max=0.08
g_max=0.08

I_f=100*2
I_g=100*2 #make these bigger if you wish

f=seq(from=f_min,to=f_max,length=I_f)
g=seq(from=g_min,to=g_max,length=I_g)
L_T_S=array(0,c(I_f,I_g))
L_T_S_norm=array(0,c(I_f,I_g))
L_P_S=array(0,c(I_f,I_g))
L_P_S_norm=array(0,c(I_f,I_g))
L_S=array(0,c(I_f,I_g))
L_S_norm=array(0,c(I_f,I_g))

#Likelihood of symptomatics in the sample
for(i_f in 1:I_f)
  {
  for(i_g in 1:I_g)
  {
    prob=f[i_f]+g[i_g]
  L_T_S[i_f,i_g]=dbinom(T_S_bar,T,prob,log=FALSE)
```

```
   mean_n=T*(f[i_f]+g[i_g])
   var_n=T*(f[i_f]+g[i_g])*(1-(f[i_f]+g[i_g]))

   L_T_S_norm[i_f,i_g]=(1/sqrt(2*pi*var_n))*exp(-(mean_n-T_S_bar)^2/(2*var_n))
  }
}


 #contours
 LL_T_S_max=max(log(L_T_S))
 criticalLevel= LL_T_S_max-2*1.96
 LL_T_S_norm_max=max(log(L_T_S_norm))


#Likelihood of positive tests P_S_bar among symtomatics in the sample
for(i_f in 1:I_f)
{
 for(i_g in 1:I_g)
  {
   prob=(f[i_f]*(1-p_SFN)+g[i_g]*p_SFP)/(f[i_f]+g[i_g])
   L_P_S[i_f,i_g]=dbinom(P_S_bar,T_S_bar,prob,log=FALSE)
  }
}

L_S=L_T_S*L_P_S

#Gausian approximation to the binomials
for(i_f in 1:I_f)
{
 for(i_g in 1:I_g)
  {
   p_sp=(f[i_f]*(1-p_SFN)+g[i_g]*p_SFP)/(f[i_f]+g[i_g])
   mean_n=T_S_bar*p_sp
   var_n=T_S_bar*p_sp*(1-p_sp)
   L_P_S_norm[i_f,i_g]=(1/sqrt(2*pi*var_n))*exp(-(mean_n-P_S_bar)^2/(2*var_n))
  }
}

L_S_norm=L_T_S_norm*L_P_S_norm


quartz() #This is Figure 8 in the main text
par(mfrow=c(1,1))
image.plot(f,g,L_S, xlab="f", ylab="g",main="Likelihood of symptomatic data")
points(f_t,g_t,col="white", pch=19, cex=1.5)
```

```
mle_indices=which(L_S == max(L_S), arr.ind = TRUE)
g_hat_index=mle_indices[2]


#now the log-likelihood  #This is not shown either in the main text or the Supplementary figures
#but is interesting to look at
LL_S_max=max(log(L_S))
criticalLevel= LL_S_max-2*1.96

quartz()
par(mfrow=c(1,1))
leg=cbind("Log Likelihood of S_bar with contours,T= ",p_d_T)
image.plot(f,g,log(L_S), xlab="f", ylab="g",main=leg)

contour(f, g, log(L_S), levels=c(criticalLevel), col="white",add=TRUE,lwd=2)
contour(f, g, log(L_S_norm), levels=c(criticalLevel), col="grey",add=TRUE,lwd=2,lty=2)
points(f_t,g_t,col="white", pch=19, cex=1.5)


#**************** Now generate results shown in Figures 9, 10, and the Supplementary
Figures
#The first step is the numerical exploration, reported in the text without figures,
#of the profile and marginal likelihoods

L_S_marg=array(0,I_f)
L_S_profile=array(0,I_f)
#prior for g
g_0=rep(1,I_g)


for(i_f in 1:I_f)
{
  L_S_profile[i_f]=L_S[i_f,g_hat_index]
  for(i_g in 1:I_g)
  {
    L_S_marg[i_f]=L_S_marg[i_f]+L_S[i_f,i_g]*g_0[i_g]
  }
}

LL_S_profile=log(L_S_profile)
LL_S_marg=log(L_S_marg)
delta_pm=max(LL_S_marg)-max(LL_S_profile)


quartz()
par(mfrow=c(1,3))
```

```
plot(f,LL_S_profile,type="l",lwd=3,xlab="f",ylab="Profile likelihood",main="Profile")
abline(v=f_t,lty=5,lwd=2)
abline(h=max(LL_S_profile)-1.96,lty=3,lwd=2)
#abline(h=max(LL_S_profile)-2*1.96,lty=3,lwd=2)
plot(f,LL_S_marg,type="l",lwd=3,xlab="f",ylab="Marginal likelihood",main="Marginal")
abline(v=f_t,lty=5,lwd=2)
abline(h=max(LL_S_marg)-1.96,lty=3,lwd=2)
#abline(h=max(LL_S_marg)-2*1.96,lty=3,lwd=2)
plot(f,log(L_S_marg),type="l",lwd=3,xlab="f",ylab="Profile likelihood",main="Profile in red")
lines(f,log(L_S_profile)+delta_pm,col=2,lwd=3)

#Conclusion:  marginal or profile give about  the same result except in the tails


#****************************** This part of the code generates the results reported in
#Figures 9, 10, and the Supplementary Figures *****

rho_hat_bar=(1/(c_2*f_hat_bar))*(d_1-b_1 )
rho_min=rho_t/3
rho_max=rho_t*3

rho_min=0.0
rho_max=4
rho_max=10 #need this for very small values of f_t or large values of rho

I_rho=300
rho=seq(from=rho_min,to=rho_max,length=I_rho)
L_P=array(0,c(I_f,I_g,I_rho))
L_P_norm=array(0,c(I_f,I_rho))  #just go directly to the profile likelihood

for(i_f in 1:I_f)
{
 for(i_g in 1:I_g)
 {
  for(i_rho in 1:I_rho)
  {
  prob=(rho[i_rho]*f[i_f]*(1-p_AFN)+(1-g[i_g]-f[i_f]*(1+rho[i_rho]))*p_AFP)/(1-f[i_f]-
g[i_g])
  L_P[i_f,i_g,i_rho]=dbinom(P_bar-P_S_bar,T-T_S_bar,prob,log=FALSE)
  }
 }
}

#Ready for the total likelihood
L_Total=array(0,c(I_f,I_g,I_rho))
L_Total_profile=array(0,c(I_f,I_rho))
```

```
LL_Total_profile=array(0,c(I_f,I_rho))
L_Total_profile_norm=array(0,c(I_f,I_rho))

for(i_rho in 1:I_rho)
{
 L_Total[,,i_rho]=L_S[,]*L_P[,,i_rho]
}

L_Total_profile=L_Total[,g_hat_index,]
LL_Total_profile=log(L_Total_profile)

L_Total_marg=array(0,c(I_f,I_rho))
LL_Total_marg=array(0,c(I_f,I_rho))

for(i_f in 1:I_f)
{
 for(i_rho in 1:I_rho)
 {
  for(i_g in 1:I_g)
  {

   L_Total_marg[i_f,i_rho] = L_Total_marg[i_f,i_rho]+L_Total[i_f,i_g,i_rho]*g_0[i_g]
 }
}
}

for(i_f in 1:I_f)
{
 for(i_rho in 1:I_rho)
 {
  p_ap_n=f[i_f]*rho[i_rho]*(1-p_AFN)+(1-g[g_hat_index]-(1+rho[i_rho])*f[i_f])*p_AFP
  p_ap_n=p_ap_n/(1-f[i_f]-g[g_hat_index])

  mean_n=(T-T_S_bar)*p_ap_n
  var_n=(T-T_S_bar)*p_ap_n*(1-p_ap_n)

   L_P_norm[i_f,i_rho]=(1/sqrt(2*pi*var_n))*exp(-(P_bar-P_S_bar-mean_n)^2/(2*var_n))
 }
}

#need the normal approximation for the profile likelihood on susceptible data
for(i_f in 1:I_f)
{
 for(i_rho in 1:I_rho)
 {
L_Total_profile_norm[i_f,i_rho]=L_S_norm[i_f,g_hat_index]*L_P_norm[i_f,i_rho]
```

```
#print(L_Total_profile)
  }
}

#plot(rho,LL_Total_profile[2,])
quartz()
par(mfrow=c(1,1))
leg=cbind("Log(Profile Likelihood) of all Data with contours, T=",p_d_T)
image.plot(f,rho,LL_Total_profile, xlab="f", ylab="rho",main=leg)
points(f_t,rho_t,col="white",pch=19)

LL_Total_profile_max=max(LL_Total_profile)

criticalLevel= LL_Total_profile_max-2*1.96
contour(f, rho, LL_Total_profile, levels=c(criticalLevel), col="white",add=TRUE,lwd=2)
contour(f, rho, log(L_Total_profile_norm), levels=c(criticalLevel),
col="grey",add=TRUE,lwd=2)



#Add the equation for rho [Eqn 28 of the main text ]
rho_fg=rep(0,I_f)
for(i_f in 1:I_f)
{
b_1=(1-f[i_f]-g_hat_bar)*p_AFP
d_1=(P_bar-P_S_bar)*(1-f[i_f]-g_hat_bar)/(T-T_S_bar)
c_2=1-p_AFN-p_AFP
rho_fg[i_f]=(1/(c_2*f[i_f]))*(d_1-b_1 )
}

lines(f,rho_fg,lwd=3,col="grey",lty=4)
#now figure out the min and max of rho
rho_contour=rep(0,i_f)

rho_min=100
rho_max=-1
f_min=0
f_max=0

#********************** To generate Figure 11, we need to find the values of f and rho
# on the contour corresponding to the maximum and minimum values of rho
# quartz()
# par(mfrow=c(1,1))
# leg=cbind("Log(Profile Likelihood) of all Data with contours, T=",p_d_T)
# image.plot(f,rho,LL_Total_profile, xlab="f", ylab="rho",main=leg)
# points(f_t,rho_t,col="white",pch=19)
```

```
#
# criticalLevel= LL_Total_profile_max-2*1.96
# contour(f, rho, LL_Total_profile, levels=c(criticalLevel), col="white",add=TRUE,lwd=3)




#Get all of the points on the contour
N_contour=5*i_f
f_contour=rep(1,N_contour)
rho_contour=rep(1,N_contour)
n_contour=0
rho_max=-1
rho_min=5

#now get the max and min of rho and of f+rho. Thanks Lisa Schwarz and Bobby Gramacy!
#use x and y for the contour plot


x=f
y=rho
contour(x, y, LL_Total_profile, levels=c(criticalLevel), col="white",add=TRUE,lwd=3)
#cout <- contourLines(x, y, LL, levels=c(criticalLevel))
contour_out=contourLines(x, y, LL_Total_profile, levels=c(criticalLevel))

ymax=max(contour_out[[1]]$y)
ymin=min(contour_out[[1]]$y)

i_ymax=which(contour_out[[1]]$y==max(contour_out[[1]]$y))
i_ymin=which(contour_out[[1]]$y==min(contour_out[[1]]$y))

#and here the x value corresponding to the max of y
f_max=contour_out[[1]]$x[i_ymax]
rho_max=contour_out[[1]]$y[i_ymax]

f_min=contour_out[[1]]$x[i_ymin]
rho_min=contour_out[[1]]$y[i_ymin]

#find the minimum and maximum values of f*rho along the contour

fxrho=contour_out[[1]]$x*contour_out[[1]]$y

fxrho_min=min(fxrho)
fxrho_max=max(fxrho)
```

```
#*********************************** Finally ready for the prize -- Figure 11
******************************
I_gs=100
gs=seq(from=1,to=100,length=I_gs)
Risk=array(0,c(5,I_gs))

#risk with the point estimate

for(i_gs in 1:I_gs)
{
  Risk[1,i_gs]=1-(1-f_hat_bar*rho_hat_bar)^gs[i_gs]

  Risk[2,i_gs]=1-(1-f_min*rho_min)^gs[i_gs]

Risk[3,i_gs]=1-(1-f_max*rho_max)^gs[i_gs]
Risk[4,i_gs]=1-(1-fxrho_min)^gs[i_gs]
Risk[5,i_gs]=1-(1-fxrho_max)^gs[i_gs]
}


for(i_gs in 1:I_gs)
{
  print(1-(1-f_hat_bar*rho_hat_bar)^gs[i_gs])

}

quartz()
leg=cbind("Risk of Infected Asymptomatic, T=",p_d_T)
plot(gs,Risk[1,],type="l",lwd=2,xlab="Group size",ylab="Risk",ylim=c(0,1),main=leg)
for(i in 4:5)
{
  lines(gs,Risk[i,],lwd=2,lty=i)
}




# #normalize in order to get 95% of the area
# L_Total_profile_norm=L_Total_profile/sum(L_Total_profile)
#
# quartz()
# par(mfrow=c(1,1))
# image.plot(f,rho,L_Total_profile_norm, xlab="f", ylab="rho",main="Normalized Profile
Likelihood of all Data")
```

```
# points(f_t,rho_t,col="white",pch=19)

# quartz()
# par(mfrow=c(1,1))
# image.plot(f,rho,LL_Total_marg-LL_Total_profile, xlab="f", ylab="rho",main="Log(Marginal
Likelihood)-Log(Profile)")
# points(f_t,rho_t,col="white",pch=19)


#************************ Now make the four panel image for PLoS XX
******************
quartz()
par(mfrow=c(2,2))
Test=T
p_d_s=toString(Tests)



plot(f_hat[1:100],xlab="Simulation number",ylab="Point estimate of f")
abline(h=f_t,lty=3,lwd=3)
abline(h=mean(f_hat),lty=3,col=2,lwd=3)
abline(h=mean(f_hat)+1.96*sd(f_hat),lty=3,col=2,lwd=2)
abline(h=mean(f_hat)-1.96*sd(f_hat),lty=3,col=2,lwd=2)

plot(rho_hat[1:100],xlab="Simulation number",ylab="Point estimate of rho")
abline(h=rho_t,lty=3,lwd=3)
abline(h=mean(rho_hat),lty=3,col=2,lwd=3)
abline(h=mean(rho_hat)+1.96*sd(rho_hat),lty=3,col=2,lwd=2)
abline(h=mean(rho_hat)-1.96*sd(rho_hat),lty=3,col=2,lwd=2)

leg=cbind("Log(Profile Likelihood), T=",p_d_T)
#remove NAs from the total
LL_Total_profile=na.omit(LL_Total_profile)
image.plot(f,rho,LL_Total_profile, xlab="f", ylab="rho",main=leg)
points(f_t,rho_t,col="white",pch=19)


plot(f,LL_Total_profile[,I_rho/2])

plot(rho,LL_Total_profile[3,])
LL_Total_profile_max=max(LL_Total_profile)

criticalLevel= LL_Total_profile_max-2*1.96
contour(f, rho, LL_Total_profile, levels=c(criticalLevel), col="white",add=TRUE,lwd=2)
contour(f, rho, log(L_Total_profile_norm), levels=c(criticalLevel),
col="grey",add=TRUE,lwd=2)
```

```
lines(f,rho_fg,lwd=3,col="grey",lty=4)

leg=cbind("Risk of Infected Asymptomatic, T=",p_d_T)
plot(gs,Risk[1,],type="l",lwd=2,xlab="Group size",ylab="Risk",ylim=c(0,1),main=leg)
for(i in 4:5)
{
  lines(gs,Risk[i,],lwd=2,lty=i)
}
```
